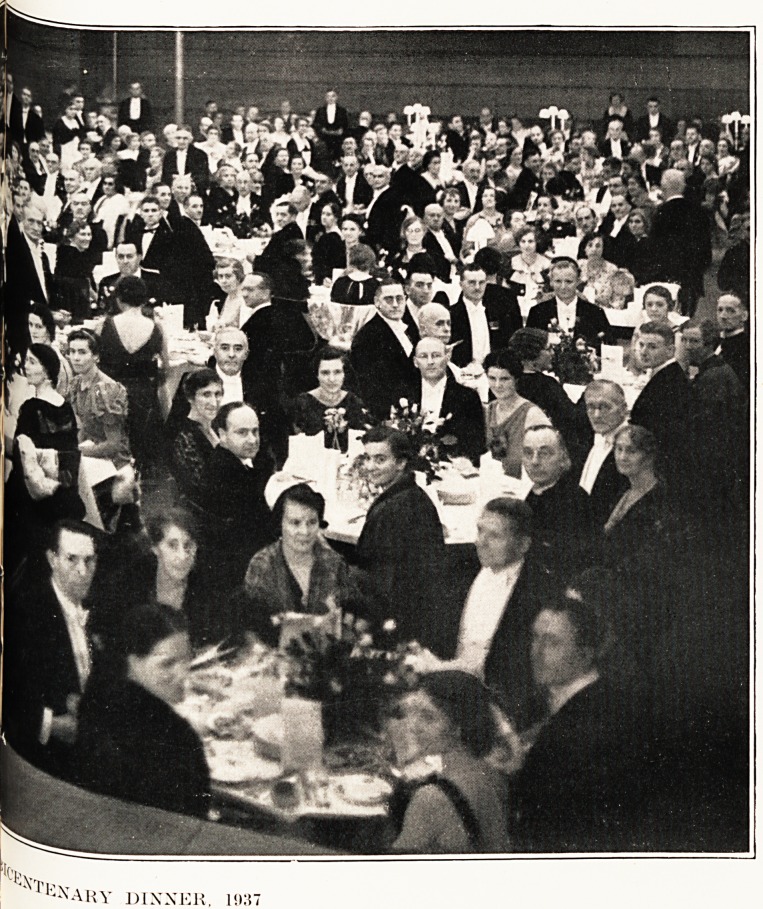# The Bristol Royal Infirmary Bicentenary
*With acknowledgements to *The Times* and *The Western Daily Press*.


**Published:** 1937

**Authors:** 


### THE BRISTOL ROYAL INFIRMARY BICENTENARY.*

---

The two hundredth anniversary of the opening of this the oldest provincial teaching hospital in the kingdom was officially celebrated on 21st and 22nd October. On the evening of 21st October the Right Honourable the Lord Mayor of Bristol and the Lady Mayoress held a reception in the Art Gallery, attended by about a thousand guests: Aldermen, Councillors, Governors, Honorary Medical Staff and supporters of the Royal Infirmary. Several features of special interest were provided, which included an exhibition of paintings, drawings and engravings of the Royal Infirmary buildings at various times and of persons connected with its early history. Amongst these were :—

Portraits of John Elbridge, the Founder; Dr. Bonython; Richard Smith and other early Physicians and Surgeons.

The Vellum Book containing lists of early subscribers, including most of the eminent Bristolians of the early eighteenth century.

The first Patients' Admission Book.

A Model of the first Building.

Drawings and Engravings showing the site before the erection of the first Infirmary.

Photographs of the opening of the Extension in 1912.

Examples of the Committee's Gold Medal, the Paul Bush and other Infirmary Medals.

The following short account of the Royal Infirmary was included in the programme which the guests received:—

About the middle of the year 1736 Dr. Bonython, a physician of Cornish extraction, who practised for many years

* With acknowledgements to *The Times* and *The Western Daily Press*.

in Park Row, near the Red Lodge, conceived a scheme to set up an Infirmary in Bristol. He printed his proposals, and a subscription book (" The Vellum Book ") was opened on 22nd November, 1736. The first General Meeting of Subscribers was held on 23rd December, 1736, when John Elbridge was appointed Treasurer. Elbridge was a New Englander. His parents had left Bristol to settle in Massachusetts, and there he was born. At the age of twelve he was sent back to Bristol, where he lived with his cousin Thomas Moore, whom he succeeded as Controller of His Majesty's Customs in Bristol. John Elbridge was a wealthy and generous benefactor, who built and furnished the Bristol Infirmary at his own sole expense. He died a year after its opening, bequeathing £5,000 to the institution. He is the acknowledged Founder of the Charity.

The Infirmary stands on a parcel of land which once belonged to the Priory of St. James. The present St. James' Church was the Chapel of that Priory which was founded in 1129 by Robert Earl of Gloucester for the black monks of the Benedictine Order.

On the 30th June, 1737, the Infirmary was quietly opened for out-patients, and the ceremonial opening took place on 13th December, 1737, when the Mayor and Corporation, the Faculty (*i.e.* the Medical Staff) and the Trustees met at the Infirmary, proceeded to Divine Service at St. James' Church, and finished the day with a dinner at the Nag's Head Tavern.

In 1700 Kingsdown was still a down. An estate called the Montagues, belonging to the Dightons (their name is recalled by Dighton Street), was bought by Charles Greville, who built on it the Montague Tavern, in 1737. He also began to build on the down a suburb, and in 1760 two newly-built houses were offered for sale, one of their advantages being that children could be sent there for the holidays " to get fresh air."

In 1749 at a Quarterly Meeting of Subscribers it was decided to place over the entrance to the Infirmary " in gold letters " the inscription " Charity Universal," and this inscription was replaced over the entrance of the new building which was opened in 1792. For by that time the first Infirmary (Elbridge's building) had proved inadequate ; it was pulled down and a new one erected of which the central wing was completed by 1792. The remainder took twenty years more to build. This is the present old Infirmary whose appearance was at a later date greatly altered by the addition of a parapet which conceals the original red-tiled, gabled roofs. Further changes were made in 1860, when the East Wing, containing the Chapel and Museum, were added, and in 1866, when the West Wing which houses the Children's Ward was erected at the expense of Mr. T. W. Hill.

In 1875, owing to its insanitary state, the Infirmary was closed for alterations, and the work of the Charity was carried on in temporary premises in Colston Street for twelve months. After this no major alterations were made to the buildings until Sir George White became President in 1904. By his efforts the Infirmary was cleared of debt, the number of annual subscribers was greatly increased, and a special fund of £50,000 was raised by the end of 1906. This fund was devoted to the erection of the new wing, which was opened on 28th June, 1912, by Their Majesties King George V and Queen Mary, as a memorial to King Edward VII. From 1914 to 1919 the King Edward VII block was occupied as a War Hospital (2nd Southern General Hospital).

The presidency of Sir George White marked a notable era in the history of the Royal Infirmary (permission to add the word " Royal " to the title of the institution had been granted by Queen Victoria in 1850). Not only was the new block added on the north side of Marlborough Street, but the Nurses' Home was largely extended, and the magnificent garden surrounding the Home was formed and thrown open to public view by demolishing the house at the foot of Alfred Hill.

At the close of the War, during the Presidency of Mr. H. H. Wills, an endeavour was made to amalgamate the Royal Infirmary and the General Hospital. The attempt was unsuccessful, and a sum of £100,000 which Mr. Wills had offered in order to finance the project was given by him to the Infirmary.

Although Mr. Wills did not live to see his wishes fulfilled, his plan of amalgamation was agreed to by the Governors of both institutions, in March, 1937. Thanks to the patient handling of the proposal by all concerned, identical resolutions were passed at meetings of the Governors of the Infirmary and Hospital requesting the Committees to take the necessary steps to amalgamate the two institutions with central management and pooling of funds. The Committees are now engaged upon formulating a scheme for giving effect to the subscribers' wishes.

Thus the Bicentenary of the Royal Infirmary marks not only two hundred years of effort for the alleviation of sickness and suffering, but also the close of over a hundred years of unprofitable rivalry.

The Museum and Art Gallery Committee arranged an exhibition of films in the Museum Lecture Theatre :—

1. The Cloud Burst and Floods in Bristol, 29th June, 1937, when two inches of rain fell in 40 minutes.

2. Civic Procession to Bristol Cathedral, Sunday, 9th May, 1937.

3. Bristol's Floral Decorations for the Coronation, May, 1937, in natural colours, depicting : Blaize Castle Nursery, College Green, Corn Exchange, Guildhall, Council House, St. Mary Redcliffe (Rush Sunday), Mansion House, Red Lodge, Victoria Rooms, Royal West of England Academy, Royal Empire Society, Museum and Art Gallery, Colston Hall, Old Market Street, Docks Office, Colston Avenue, Night Illuminations.

These natural colour films were a revelation to most of the audience of the progress that has been achieved in this direction.

The City of Bristol Police Light Orchestra provided the following programme of music :—

| | | | |
|---|---|---|---|
| 1. | March .. | .. " Aguero " .. | .. *Franco* |
| 2. | Valse .. | .. " Narenta " .. | .. *Komzak* |
| 3. | Serenade | .. " Moontime " .. | .. *Collins* |
| 4. | Ungarische Tanze No. 1. .. | .. | .. *Brahms* |
| 5. | Chanson Indoue | .. .. .. | *Rimsky-Korsakov* |
| 6. | Valse .. | .. " Emperor " .. | .. *Strauss* |
| 7. | Selection | " The Student Prince " | .. *Romberg* |
| 8. | Chanson | .. " In Love " .. | .. *Friml* |
| 9. | Selection | .. " Lilac Time " .. | *Schubert-Clutsam* |
| 10. | Valse .. | " Gold and Silver " | .. *Lehár* |
| 11. | Selection from Sullivan's Works .. | | .. *Arr. Godfrey* |
| 12. | Humoreske | .. .. .. .. | .. *Dvořák* |
| 13. | Toreador et Andalouse | .. .. | .. *Rubenstein* |
| 14. | Selection | .. " Bitter Sweet " .. | .. *Coward* |
| 15. | Valse .. | " Die Hydropaten " | .. *Gung'l* |

GOD SAVE THE KING.

---

Director of Music .. .. Captain F. W. WOOD, M.V.O.

The Lord Mayor, in welcoming his guests, referred to the continuous and happy association between the City authorities and the Infirmary throughout the latter's existence, for the formal opening two hundred years before was attended by the then Mayor and Aldermen. He felt assured that the new developments in the provision of medical services in the City would continue to exhibit the same happy spirit of co-operation.

On Friday, 22nd October, *The Times* printed an account, from which the following is taken :—

In the autumn of 1737 the Bristol Infirmary, now called the Bristol Royal Infirmary, was opened for the reception of in-patients. It is believed that, outside London, this was the first hospital in the country to receive in-patients.

The state of the sick poor in the eighteenth century cried aloud unto Heaven. Poverty, overcrowding, ignorance, and the lack of public sanitation were terrible. Most large towns had a pauper institution—that in Bristol being called St. Peter's Hospital—to which persons could be taken if they were homeless and very ill, but for the most part the poor were dependent for treatment on the almost gratuitous service of apothecaries, who were often quite uneducated. A leading part in the foundation of the Infirmary was taken by John Elbridge, Richard Champion and Dr. John Bonython, who became the first physician. It seems that the original building was built and furnished at the sole expense of John Elbridge. He was born in Massachusetts of British parents, came back to Bristol in childhood, and became Controller of His Majesty's Customs.

### SURGICAL CURIOSITIES.

The Infirmary has been singularly fortunate in its chroniclers. Richard Smith, jun., surgeon from 1796 to 1843, had a passion for collecting. He collected specimens to found a museum, still in regular use for the instruction of students, and placed in it not only a great number of specimens illustrating surgical pathology, but curiosities such as the skeletons of persons hanged in Bristol for crime, whose bodies were handed over for dissection. All the records of one such trial are preserved bound in the victim's own skin. There is also in his museum a femur, which is reputed—though no doubt the claim will be challenged—to be from the first amputation at the hip joint in England. The patient was one Sebastian de Chamours, of the Chasseurs Britanniques, who sustained a bullet wound of the thigh in the Napoleonic Wars, and evidently languished a long time with necrosis of the bone. Whether the unfortunate Frenchman lived or died there is no evidence to show. Richard Smith also rescued various garments and remains of Patrick Cotter, the Irish giant; he could not get the actual skeleton because the giant, warned by the fate of his predecessor who fell into the hands of John Hunter and who now occupies a glass case at the Royal College of Surgeons in London, had himself buried in 12 ft. of solid rock under a Roman Catholic chapel. Smith also collected innumerable details, of a chatty kind, about everybody and everything connected with the early history of the institution. All his wealth of information, gay and sober, lay almost unheeded until twenty years ago, when Munro Smith, another Infirmary surgeon, compiled his *History of the Bristol Royal Infirmary*. Probably no other hospital in the country has been the subject of such a chronicle, with such plentiful information, and related with so much gaiety.

### Great Names.

It must be admitted that changes for the better, the introduction of anaesthetics, of flap methods of amputation, of antiseptic wound treatment, and other more recent innovations, usually met with mulish opposition from a stubborn minority, and sometimes a resignation or two by those who thought the old was better. Stories survive of the hardships of those times, but behind it all there has been a record of 200 years of human kindness—usually of skilled kindness—of lives without number saved and made useful again, and of steady progress, making available even for the poorest all the resources of every advance in medical treatment. There have been great names on the roll of the honorary staff: James Cowles Prichard, the ethnologist; William Budd, probably the greatest pioneer among British epidemiologists; Greig Smith, one of the fathers of abdominal surgery in Britain.

To-day the hospital has 400 beds; the number of in-patients annually treated is 10,305 and of out-patients 70,570. It is a remarkable fact that nearly one-quarter of the babies born in Bristol are brought into the world under the care of the indoor or outdoor services of the Royal Infirmary.

In the afternoon many former students and nurses were entertained at the Infirmary by the President and Committee, and had an opportunity of visiting the buildings and seeing what improvements had been made, particularly in the development of the Fracture Department and the provision of sun roofs and balconies.

### Dinner at University Union.

The culmination of the celebrations was a dinner at the University Union—in strict accordance with eighteenth-century precedent. Besides the high table, tables were arranged for parties of various sizes, and among the 339 guests present were representatives of the religious, civic, social, professional and commercial life of the city and district. Lady (Vernon) Wills had very generously assumed responsibility for the decorations, and the masses of rosebuds which adorned every table produced a really charming effect: there was universal regret that owing to indisposition the donor was herself prevented from attending.

Supporting the President of the Royal Infirmary, Colonel P. G. Robinson, D.S.O., at the high table were the Right Honourable the Lord Mayor and Lady Mayoress of Bristol (Mr. A. F. Moon and Miss Kathleen Moon), the Sheriff (Lieut.-Comdr. Vivian J. Robinson) and the Hon. Mrs. Robinson, the Right Reverend the Lord Bishop of Bristol (Dr. C. S. Woodward), the Bishop of Clifton (Monsignor W. Lee), the Mayor and Mayoress of Bath (Mr. W. F. Long and Mrs. Long), Alderman Frank Sheppard (Chairman of the Royal Infirmary), Mr. Herbert M. Baker (Chairman of the

General Hospital), Mrs. P. G. Robinson, the Dean of Bristol (the Very Rev. H. W. Blackburne), Colonel Dan Burges (Master of the Merchant Venturers) and Mrs. Burges, Dr. T. Loveday (Vice-Chancellor of Bristol University) and Mrs. Loveday, His Honour Judge Wethered and Miss Wethered, Professor R. J. Brocklehurst (Dean of the Medical Faculty of the University) and Mrs. Brocklehurst, Professor E. Fawcett, F.R.S., Professor and Mrs. H. J. Drew Smythe, Professor and Mrs. C. B. Perry, Professor and Mrs. S. E. Whitnall, Dr. E. C. Morland (Editor of *The Lancet*), Professor R. H. Parry (Medical Officer of Health), Major E. Cadbury (Treasurer of the Infirmary), E. C. Smith, Esq. (Secretary and House Governor) and Miss E. Johnston (matron).

Dr. and Mrs. P. Watson-Williams, Dr. and Mrs. R. Charles, Professor and Mrs. J. A. Nixon, Dr. and Mrs. R. C. Clarke, Professors A. Rendle Short and the Honorary Medical Staff of the Infirmary.

The hospitals of Great Britain were represented by Dr. C. E. Purslow, M.D., M.R.C.P., R. Beatson Hird, Esq., F.R.C.S. (Birmingham Royal Infirmary) ; Dr. Ivor J. Davies, M.D., F.R.C.P., Professor Lambert C. Rogers, F.R.C.S., F.R.A.C.S. (Cardiff Royal Infirmary) ; Miss E. E. P. MacManus, O.B.E., Dr. H. C. Cameron, M.D., F.R.C.P. (Guy's Hospital) ; Dr. C. Wilfrid Vining, M.D., F.R.C.P. (Leeds Royal Infirmary) ; R. Stopford - Taylor, Esq., D.S.O., F.R.C.S., Captain William Rutter (Liverpool Royal Infirmary) ; Professor L. Stanley Dudgeon, C.M.G., C.B.E., F.R.C.P., Mr. J. Herbert Scrutton (St. Thomas's Hospital) ; A. W. Fawcett, Esq., F.R.C.S. (Sheffield United Hospital).

Edinburgh Royal Infirmary, The London Hospital, Manchester Royal Infirmary and St. Bartholomew's

BRISTOL ROYAL INFIRMARY

[illegible]ICENTENARY DINNER, 1937

Hospital were unable to send representatives, but offered congratulations on the occasion and sincere good wishes for the success of the celebrations.

Alderman Frank Sheppard proposed "The City and County of Bristol." He said Bristol was rich in charities and historical associations, but generally its citizens knew all too little of them. He spoke warmly of the way Mr. A. F. Moon had carried out his duties as Lord Mayor, and remarked that it was a fine characteristic of English local government and politics that men of opposing views could retain their friendship.

Alderman Sheppard said that he had the greatest admiration for those responsible for running the Royal Infirmary, with which he had been associated for twenty years. The city was very proud of its work, and so long as such institutions could command the same devoted service as in the past, there need be no fear for their future. They were thankful that there were men of ability ready to give their best to the sick poor of the city.

The Lord Mayor responded, and proposed the "Bristol Royal Infirmary."

He said that if young people to-day would give as much of their time to public service as Alderman Sheppard had, there need be no anxiety for the city's future.

"It is the standard of her people that makes a city great," said the Lord Mayor, "and we are all trying to make that standard as high as possible."

He said no one had a greater regard for work of the Infirmary than he had, and he paid his special tribute to the nurses, "with their charming presence and cheerful smiles."

Speaking of the constant support necessary to maintain the work of a great hospital, he said there had been men of splendid benevolence in the past, and to-day it was a matter for gratitude there were some following in their steps.

Colonel P. G. Robinson, in reply, said how sorry they were that the illness of the Duchess of Beaufort prevented the Duke from attending, and remarked that the Duke's family had been directly associated with the Infirmary for something like 160 years.

He contrasted the first year of the Infirmary's existence, when 194 in-patients and 234 out-patients were treated, with last year, when the corresponding numbers were 10,300 and 70,500. The first year's expenditure was £434 and last year was £72,000.

Happily the Royal Infirmary had always kept abreast of changing conditions.

"To-night," he said, "we are at the birthday party of a serene old lady born in the time of George II, and we are here to celebrate, not her antiquity, but the fact that she has been able to go on with increasing usefulness for 200 years. Indeed, she is so young that she is even contemplating matrimony.

"I cannot say whether she is the wooer or the wooed, or whether it is a love match or a marriage of convenience . . . but I can tell you that all her friends are confident of the happy outcome of this rather tardy adventure."

The President went on to announce that three benefactors of the Royal Infirmary had come forward with generous gifts to mark the occasion: Mr. S. H. Justin had given them £1,500; the executors of Mr. Samuel White had given £1,050; their treasurer, Major E. Cadbury, had given £1,000.

In thanking the benefactors, the President ventured to hope that others would follow their good example!

Mr. A. Rendle Short, senior surgeon at the Royal Infirmary, responded on behalf of his medical colleagues.

He said that 200 years ago the condition of the sick poor in Bristol, as elsewhere, was deplorable in the extreme. In those circumstances, medical men and business men united in pioneer work to serve the community, and they blazed a trail that had been happily maintained ever since.

During half the period of the Infirmary's history surgery had been performed without chloroform anæsthesia.

"In those days," said Mr. Rendle Short, "operations were great occasions. Newspaper accounts used to read: 'Yesterday Mr. Richard Smith cut for the stone at the Bristol Infirmary in the presence of the Mayor and several gentlemen.'"

The surgeon's record book for 1875 showed operation after operation leading to disaster, often after the most trivial surgical procedures. Shortly after the introduction of the antiseptic treatment those horrors diminished.

A rhyme related about Dr. J. C. Prichard, the ethnologist, ran:—

> "He leaves his patients full of sorrow—
> They must be cupped to-day and bled to-morrow."

At another period the cellars of Bristol were said to be scoured clean for cobwebs which a leading physician used to prescribe for his patients.

Mr. Rendle Short wondered if fifty years hence treatments favoured to-day would appear equally grotesque.

He referred to the pioneer work which had been done at the Royal Infirmary in many branches of medicine and surgery, and said it had also become an important training ground for medical students and nurses.

There was a time, however, when the senior resident officer used to stand in the lodge to see that the one and only night nurse was sufficiently sober to go on duty! But by degrees the nursing service had vastly improved. There was more kindness in the treatment of hospital patients now than there was even so comparatively recently as the beginning of his own experience. Although two hundred years old, the Royal Infirmary was by no means senile: he had never known a time when there were so many visions of further progress. The best medical service in the city could be provided by team work, which would afford first-class service in every branch of medicine and surgery. For this the city required a single medical centre, serving alike the voluntary hospitals and the municipal hospitals. In conclusion, he would remind his hearers how very deeply they were indebted to the little Committee who had arranged the Bicentenary Celebrations and especially to the Secretary, Mr. Eric Watson-Williams, who had been responsible for the organization of the dinner that evening.

Dr. P. Watson-Williams as senior member of the Royal Infirmary Faculty proposed the toast of "The Guests." He paid a tribute to the Lord Mayor and Lady Mayoress, and those who represented every aspect of religious, civic and social activity in Bristol and the neighbourhood, and went on to voice the very hearty and sincere thanks of the Infirmary to the delegates from British hospitals for the pains they had taken in coming long distances to do honour to the occasion. It was often helpful to have the criticisms from those without. When some years ago, with a party of the British Medical Association, he visited Toronto and the United States, they had many questions to answer as to their impressions of the country they were visiting. One of the party, with more temerity than discretion, asked a charming hostess what had struck her most about us. "Well," she replied, "if I may say so, it is your very strong British accent."

The time was drawing to a close, so he recalled to mind the old Sussex saying, "Every time a sheep baas, he misses a tuft of grass," and he would be brief, as they hoped for many tufts of grass from those who would respond to the toast of our guests.

He had been attached to the Royal Infirmary for more than half a century, when one had no X-rays, no bacteriology, and cases of typhoid fever and diphtheria were not excluded from the general wards. What strides and advances we had made in so many directions!

Of the many movements of thought and problems ripe for research one was how to grapple with the mental defective. But another was the equally important question of the causal relationships between disease and moral delinquency and crime. Even as far back as the thirteenth century St. Thomas Aquinas envisaged this and enjoined that punishment should be always medical.

If at length we are to take the most judicious steps in bringing these problems to a practical and wise solution science wanted the guidance and active collaboration of all that spiritual aspect of life which our Lord Bishop stood for.

The Bishop of Bristol responded in a speech of memorable urbanity and wit which his audience received in a manner that testified their appreciation ; and Miss MacManus also spoke, receiving an ovation when she rose which amply demonstrated how affectionately she is remembered in Bristol.

## Figures and Tables

**Figure f1:**
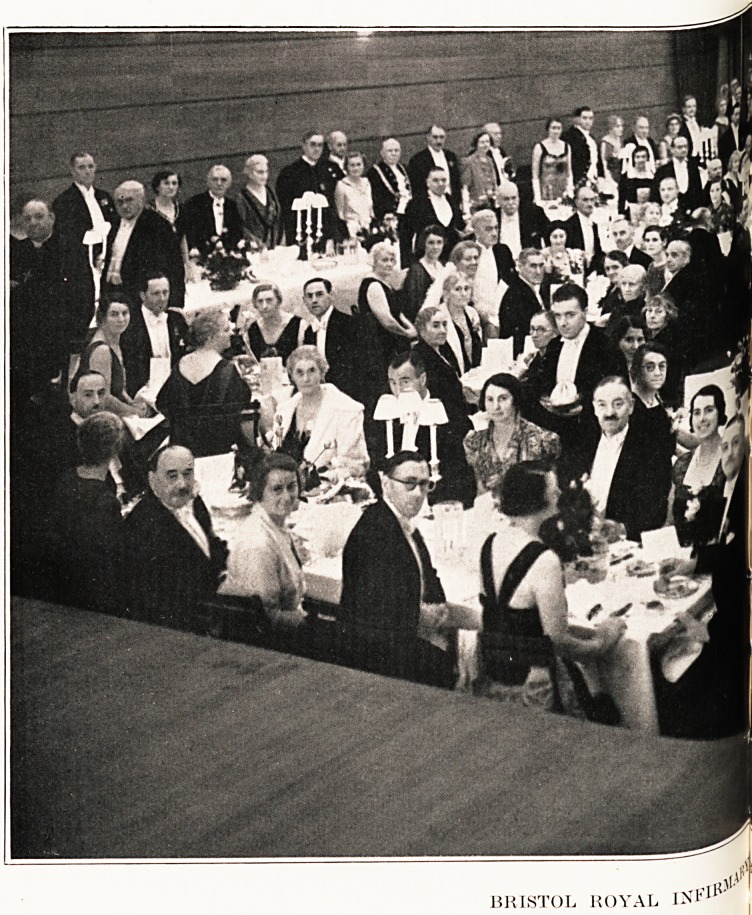


**Figure f2:**